# Genomic Characterization of a Nalidixic Acid-Resistant *Salmonella* Enteritidis Strain Causing Persistent Infections in Broiler Chickens

**DOI:** 10.3389/fvets.2021.725737

**Published:** 2021-09-01

**Authors:** Grayson K. Walker, M. Mitsu Suyemoto, Dawn M. Hull, Sesny Gall, Fernando Jimenez, Laura R. Chen, Siddhartha Thakur, Rocio Crespo, Luke B. Borst

**Affiliations:** Department of Population Health and Pathobiology, College of Veterinary Medicine, North Carolina State University, Raleigh, NC, United States

**Keywords:** *Salmonella* Enteritidis, poultry, genomics, antimicrobial resistance, broilers

## Abstract

Virulent strains of *Salmonella enterica* subsp. *enterica* serovar Enteritidis (SE) harbored by poultry can cause disease in poultry flocks and potentially result in human foodborne illness. Two broiler flocks grown a year apart on the same premises experienced mortality throughout the growing period due to septicemic disease caused by SE. Gross lesions predominantly consisted of polyserositis followed by yolk sacculitis, arthritis, osteomyelitis, and spondylitis. Tissues with lesions were cultured yielding 59 SE isolates. These were genotyped by Rep-PCR followed by whole-genome sequencing (WGS) of 15 isolates which were clonal. The strain, SE_TAU19, was further characterized for antimicrobial susceptibility and virulence in a broiler embryo lethality assay. SE_TAU19 was resistant to nalidixic acid and sulfadimethoxine and was virulent to embryos with 100% mortality of all challenged broiler embryos within 3.5 days. Screening the SE_TAU19 whole-genome sequence revealed seven antimicrobial resistance (AMR) genes, 120 virulence genes, and two IncF plasmid replicons corresponding to a single, serovar-specific pSEV virulence plasmid. The *pef*, *spv*, and *rck* virulence genes localized to the plasmid sequence assembly. We report phenotypic and genomic features of a virulent SE strain from persistently infected broiler flocks and present a workflow for SE characterization from isolate collection to genome assembly and sequence analysis. Further SE surveillance and investigation of SE virulence in broiler chickens is warranted.

## Introduction

*Salmonella enterica* subsp. *enterica* is comprised of over 2,500 serovars, including the pathogenic *Salmonella enterica* subsp. *enterica* serovar Enteritidis (SE) (1). As it relates to poultry production, SE is best known for its ability to survive on and within eggs despite causing no discernable disease in infected layers (2, 3). Because of these adaptations, SE is likely the most frequent cause of human salmonellosis globally (4), and investigations of SE today are largely focused on reducing foodborne illness. As such, the virulence features of present-day SE strains that initially colonize broilers have received relatively little attention. A fundamental understanding of SE genotypes and virulence mechanisms is essential to developing surveillance, treatment, and control approaches for broiler flocks.

*Salmonella enterica* subspecies *enterica* associated with poultry are assigned to two categories based on motility and host adaptability. First, *Salmonella enterica* subspecies *enterica* serovar Gallinarum contains two closely-related biovars, Gallinarum and Pullorum, which cause fowl typhoid and pullorum disease, respectively (1). These biovars are adapted to avian hosts, are non-motile due to mutations in flagellar genes, and have been eradicated in many developed countries but are still a significant cause of disease in countries with developing poultry production systems (5, 6). The second category holds the motile, non-host adapted serovars, including SE, which cause paratyphoid disease in poultry and encompass all foodborne *Salmonella* serovars. While this is the most common group associated with poultry in the present day, these serovars rarely cause symptomatic disease in older birds (1). Another subspecies, *Salmonella enterica* subspecies *arizonae*, causes an economically important disease in turkey production (7) but rarely affects broiler-type chickens (1).

In young broilers, SE is the common etiological agent of paratyphoid disease resulting in production losses (8). *Salmonella enterica* subsp. *enterica* serovar Enteritidis infection of young chicks is more likely to result in observable disease during natural infection and in challenge models whereas older birds are typically asymptomatic (8–10). *Salmonella enterica* subsp. *enterica* serovar Enteritidis can persistently colonize asymptomatic broiler and layer chickens from hatch to maturity (11, 12). Thus, a significant challenge encountered when attempting to control SE in broiler operations is egg-associated vertical transmission from breeders to broilers (13, 14). It is likely that SE can initially colonize chicks and poults during hatching and persist until opportunistically causing clinical disease. For example, an SE outbreak of broiler breeder origin caused fibrinous arthritic lesions of broilers aged 12 days (15). Similar lesions attributed to SE have also been observed in mature turkeys (16). *Salmonella enterica* subsp. *enterica* serovar Enteritidis is a significant threat to poultry production due to its potential to be vertically transmitted, persist, and cause opportunistic infections (15).

*Salmonella enterica* subsp. *enterica* serovar Enteritidis genetic characterization and surveillance can be achieved with a myriad of diagnostic and molecular methods, including plasmid analysis, pulsed-field gel electrophoresis, and detection of AMR and virulence genes (17, 18). With recent advances in whole-genome sequencing (WGS) efficiency and accessibility with concurrently decreasing costs, sequencing has replaced such methods as the standard for *Salmonella* surveillance, especially SE, in many countries (19–21). Additionally, WGS analysis of SE genomes can identify virulence and resistance features of field isolates, consequently adding more depth to SE genotypes. These include using mass screening software packages to identify known virulence factors and antimicrobial resistance (AMR) genes (22).

Additional identification and characterization of SE virulence features in avian hosts is warranted, especially in the context of observed clinical disease. This report describes a SE strain that not only caused disease throughout a typical 57 days growing period but was detected in two flocks grown a year apart in the same facility. Due to disease severity in broilers and subsequent isolation of SE from lesions, the aim of this study was to identify virulence and resistance features of the SE strain. After establishing lethality to broiler embryos, this strain's whole-genome sequence was screened for virulence, plasmid, and resistance genes. As virulent SE strains continue to circulate in animal and human populations, surveillance and genomic characterization are required for further virulence investigation and development of treatment and control strategies.

## Materials and Methods

### Flock Rearing

Broiler flocks A and B were reared 1 year apart (February to April; 2019 and 2020) for teaching and research at the North Carolina State University College of Veterinary Medicine. Approximately 5,000 mixed-sex Ross 708 broiler chicks were placed on the day of hatch on pine shavings that top-dressed a dirt surface and reared to 57 days. The 508 m^2^ fan ventilated, curtain-sided rearing facility included radiant brooders, forced air heaters, and paired feeder and water lines. Integrator-supplied feed containing a coccidiostat was provided with municipal water *ad libitum*.

### Sample Collection and Preliminary Characterization

Dead broilers consisting of flock mortality and culled birds were collected twice daily for necropsy. The total number of birds submitted for postmortem examination was 357 for flock A and 274 for flock B. Septicemic bacterial infections were diagnosed based on clinical signs, postmortem findings (23), and diagnostic culture results of representative birds. Cultures were collected from samples of gross lesions, including yolk sac contents, pericardial fluid, whole spleen, liver surface, joint exudate, and caseous material from inflammatory lesions in the free thoracic vertebra. Additionally, a random sample of 24 apparently healthy broilers was selected from broiler flock A at 6 days of age for yolk sac culture. Samples were collected aseptically with Stuart swabs (BD 220144), which were transported directly to an on-site laboratory and cultured on trypticase soy agar with 5% sheep blood (BD 221261), MacConkey agar (BD 212123), Columbia CNA agar with colistin and nalidixic acid (BD 221353), and XLT-4 agar (BD 223420) followed by incubation at 37°C for 18–24 h. Colonies that did not ferment lactose on MacConkey agar and appeared with a dark black center on XLT-4 were presumptively identified as *Salmonella* spp. and further purified on non-selective media. *Salmonella* spp. was confirmed by either the Biolog GenIII Microplate™ microbial identification system as described by Borst et al. (24) or PCR for the *Salmonella*-specific *invA* gene sequence (25). All isolates were then genotyped using repetitive element-based PCR methods using the (GTG)_5_ primer as described by Walker et al. (26). All isolates had identical banding patterns, suggesting clonality. This was confirmed for 15 selected isolates from broiler flock A by single nucleotide polymorphism (SNP) phylogenetic analysis using WGS of sequenced isolates (below). A representative clone, SE_TAU19, that was isolated from the yolk sac of a chick from broiler flock A, was used for all downstream experiments. The minimum inhibitory concentration of 27 antimicrobial drugs was determined by microbroth dilution methods according to guidelines defined by the National Antimicrobial Susceptibility Monitoring System (NARMS) (26). These drugs included 14 antimicrobials tested by NARMS for Gram-negative pathogens (ThermoFisher CMV3AGNF) and 13 drugs specific for treatment of poultry (ThermoFisher AVIAN1F). The breakpoints for drugs in the poultry-specific formulary (clindamycin, erythromycin, novobiocin, penicillin, and tylosin tartrate) to which *Salmonella* spp. are known to be intrinsically resistant were not interpreted (27).The strain was designated as resistant or susceptible based on available breakpoint data from the Clinical and Laboratory Standards Institute (28). The only existing breakpoint specific for poultry is enrofloxacin (29). Otherwise, human breakpoints were used for interpretation of the data.

### Embryo Lethality Assay

Fertile broiler eggs were obtained from a commercial hatchery and incubated under standard conditions (37.5°C, >50% relative humidity) to 12 days. After confirmation of viability, eight embryos were challenged via the allantoic cavity with approximately 200 CFU of SE_TAU19 as previously described (30). A positive control group of 21 embryos was challenged with the same dose of an avian pathogenic *Escherichia coli* (APEC) isolate, EC_06YS which is known to be virulent to broiler embryos from an unrelated study (31). Survivability was determined by candling eggs every 12 h. Dead embryos were individually cultured to verify presence of the challenge strain. A negative control group of six eggs injected with sterile PBS was also included in the experiment. All negative control eggs survived and were cultured upon termination of the experiment to verify sterility.

### Whole Genome Sequencing, Assembly, and Typing

Genomic DNA of 15 SE isolates collected from broiler flock A was isolated with the Qiagen DNeasy Blood and Tissue kit (Qiagen 69504). After assessing the quality of isolated DNA with a Nanodrop 2000 spectrophotometer, double stranded DNA was quantified using a Qubit 4.0 Fluorometer (ThermoFisher Scientific, Waltham, MA) with a dsDNA high-sensitivity assay kit (ThermoFisher Q32851). DNA were sequenced on an Illumina MiSeq according to the manufacturer's instructions using manufacturer supplied reagents. The Nextera DNA Flex Library Prep kit (Illumina 20015828) was used to prepare pooled libraries with an insert size of ~350 bp prior to sequencing with a MiSeq Reagent Kit v2 (500 cycles, Illumina MS-102-2003). Genome assembly and quality check were completed with CLC Genomics Workbench (Cambridge, MA) using the *de novo* assembly procedure. A minimum contig length of 200 bp was specified in the parameters and scaffolding of paired-end reads was performed. The assembled genome sequence of SE_TAU19 was uploaded to SeqSero version 1.0, a web-based *Salmonella* serotyping tool (32). The assembled genome of SE_TAU19 was blasted in CLC Genomics Workbench for the SE-specific *sdf* (*Salmonella* difference fragment) sequence for serotype confirmation (33). Multilocus sequence typing (MLST) was conducted by uploading the assembled genome to an online database (https://cge.cbs.dtu.dk/services/MLST/) (34). Clonality was confirmed through an online SNP-calling pipeline for WGS, CSIPhylogeny (https://cge.cbs.dtu.dk/services/CSIPhylogeny/) (35), with a minimum Z-score of 1.96 and default parameters described by Wuyts et al. (36) (data not shown). The SE_TAU19 sequence was originally deposited in GenBank under the alias LB_5320 (accession number AADSUW000000000.1) as part of the FDA GenomeTrakr project (Bioproject PRJNA293224).

### Identification of Virulence, Plasmid, and Resistance Genes

For additional analysis, sequencing reads were assembled and analyzed in a separate pipeline than described above. A draft genome of SE_TAU19 was generated by inputting its SRR run number (SRR8943529) in the SPAdes version 3.0-based software Shovill 1.0.4 assembler (37, 38). Default parameters were used with minimum contig length set at 200 bp and minimum contig coverage set at 5.00. The resulting contigs were screened for plasmids, virulence, and AMR genes using the following databases in ABRicate (39): Comprehensive Antimicrobial Resistance Database version 3.0.2 (CARD) (40), Virulence Factor Database (VFDB) (41), and PlasmidFinder version 2.1 (42). The assembled genome was uploaded to the ResFinder version 4.1 online database to detect chromosomal point mutations (https://cge.cbs.dtu.dk/services/ResFinder/) (43). Only sequence hits with 100% coverage and ≥98% gene identity were included. Remaining sequence hits are provided as Supplementary Material (Supplementary File 1). The plasmid sequence was assembled using plasmidSPAdes (44) with the same approach and parameters used for the genome assembly and screened independently using the above databases to identify plasmid-specific virulence genes. The presence of the detected plasmid in SE_TAU19 was confirmed by PCR amplification of a 312 bp fragment of the *spv*A plasmid virulence gene (45, 46) using primers: *spv*A-F (CAG CCT GAT GGT GGT TAA TGA) and *spv*A-R (CAG TGC TTC AAA TGG CGT ATA G). Pathogenicity in human hosts was predicted using the online database, PathogenFinder version 1.1 (https://cge.cbs.dtu.dk/services/PathogenFinder/) (47). The stepwise procedure used to characterize SE_TAU19 in this study is summarized in Figure 3.

### Statistical Analysis

Kaplan-Meier survival curves were used to determine embryo survivability, which was analyzed by the log-rank (Mantel-Cox) test of significance (GraphPad Prism version 9, GraphPad Software, San Diego, California USA).

## Results

### Clinical Observations and Antimicrobial Susceptibility

**Figure 1:**
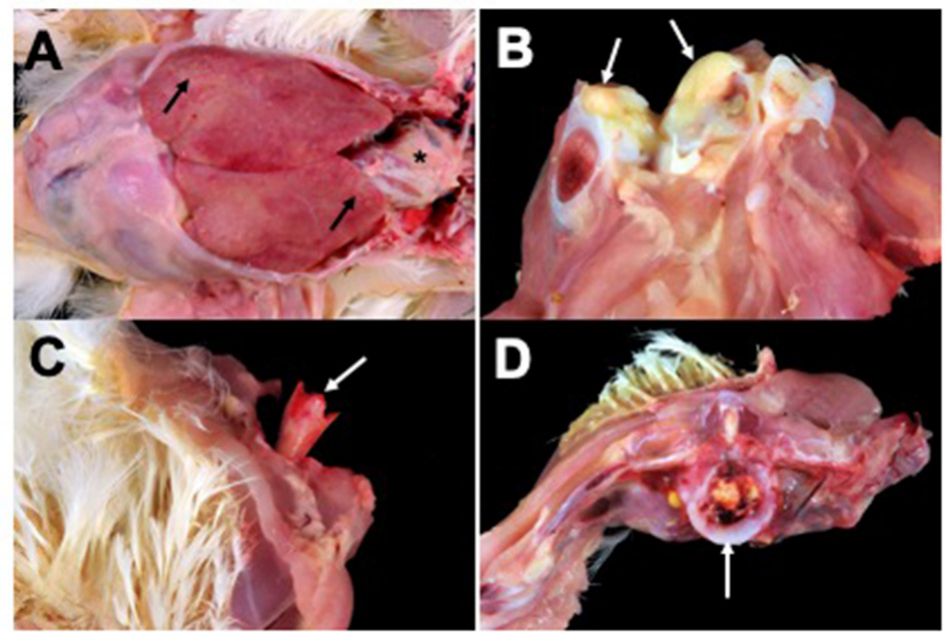
Broiler gross lesions from day 33 mortality attributed to septicemic disease caused by *Salmonella* Enteritidis (SE) strain SE_TAU19. **(A)** Fibrinous pericarditis (asterisk) and multifocal necrotizing and heterophilic hepatitis (black arrows). **(B)** Hock joint, fibrinous arthritis (white arrows). **(C)** Bacterial chondronecrosis with osteomyelitis (femoral head necrosis) (white arrow). **(D)** Vertebral osteomyelitis (white arrow).

Lesions from affected birds included evidence of septicemia (yolk sacculitis, splenomegaly, fibrinous pericarditis, and multifocal necrotizing and heterophilic hepatitis), and musculoskeletal infection (Figure 1). Based on culture results from daily necropsy, it was estimated that 37/357 (10%) and 42/274 (15%) of total mortality for flocks A and B was attributable to SE infection and a total of 59 isolates were collected from the flocks. Yolk sac samples from apparently healthy birds at 6 days in broiler flock A revealed that 5/20 harbored SE. Additional causes of mortality were attributed to heat stress during late growing. Turkey flocks reared 5 months prior to broiler flocks in the same facilities under similar conditions did not experience elevated mortality and SE was not isolated during routine organ cultures of deceased birds (data not shown). SE_TAU19 was resistant to 2 of 27 drugs tested: nalidixic acid, a quinolone, and sulfadimethoxine, a sulfonamide. The strain was classified as susceptible to the fluoroquinolone ciprofloxacin (MIC = 0.25 ng/μl).

### Embryo Lethality Assay

**Figure 2:**
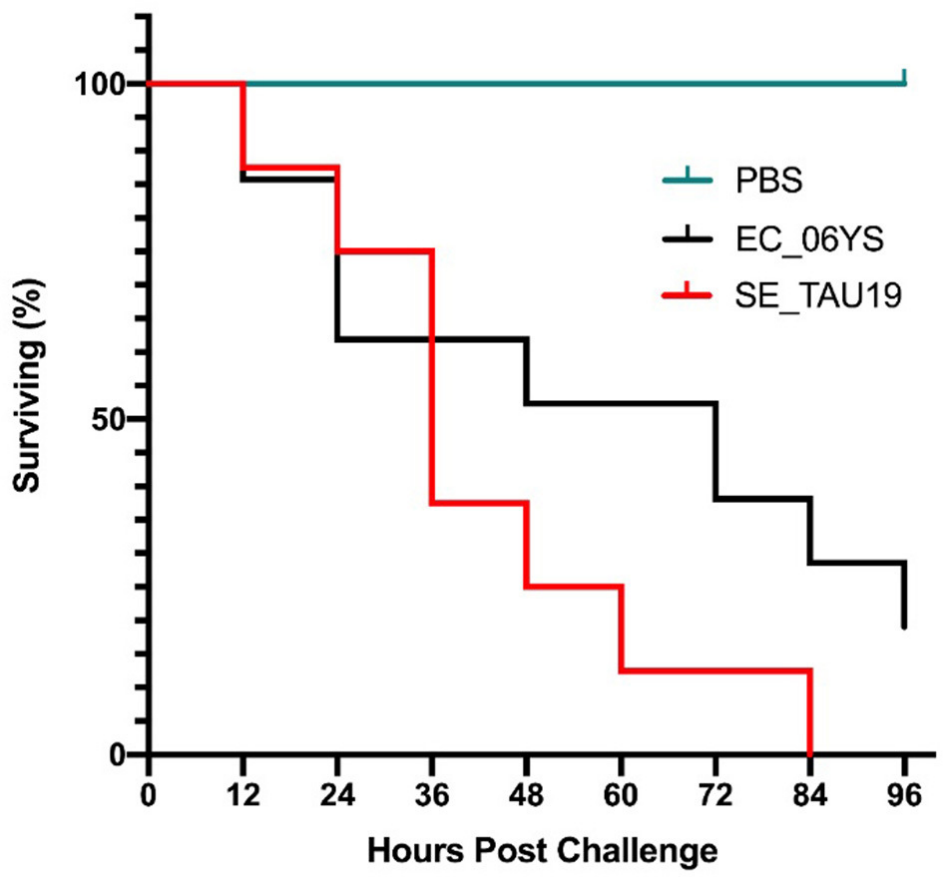
Survival of broiler embryos after challenge with *Salmonella* Enteritidis strain SE_TAU19. An avian pathogenic *E. coli* strain known to be virulent to broiler embryos, EC_06YS, was used to challenge 21 eggs and served as a positive control. Eight broiler embryos were challenged with SE_TAU19. Eggs were inoculated with approximately 200 CFU/egg via the intra-allantoic route at 12 days of incubation and candled every 12 h to monitor survivability. All embryos challenged with SE_TAU19 were dead by 84 h, while the six PBS-injected control eggs survived and EC_06YS achieved expected lethality.

When broiler embryos were challenged with SE_TAU19 at 12 days, over half died within 2 days, and all embryos were dead by 3.5 days post-injection (Figure 2). Embryo lethality due to infection with SE_TAU19 was similar to the virulent EC_06YS APEC positive control strain (*P* ≤ 0.10), which was classified as virulent based on a scheme developed by Wooley et al. (48).

### Genome Assembly and Genotypic Characterization

Sequencing of the SE_TAU19 genome included 1,106,258 reads with 56X coverage. The *de novo* assembly procedure in CLC Genomics Workbench produced 57 contigs (excluding scaffolded regions) with an average length of 82,512 bp and an N_50_ of 304,422 bp. SE_TAU19 belongs to ST-11 based on MLST typing. SeqSero and *in silico sdf* gene detection confirmed the Enteriditis serotype. Screening of the SE_TAU19 genome assembly generated by Shovill using ABRicate resulted in a total of 189 gene hits: 40 genes, 2 plasmid replicons, and 147 virulence genes (Supplementary File 1). Using a threshold of 100% coverage and 98% identity, 7 AMR genes, 2 plasmid replicons, and 103 virulence genes met criteria for inclusion (Table 1). Antimicrobial resistance genes consisted of the *aac(6*′*)-ly* chromosomally encoded aminoglycoside resistance gene, the *mds* multidrug and metal efflux pump with its regulator, and an additional multidrug and toxic compound transporter, *mdtK*. Point mutation analysis detected a guanine to thymine substitution in the *gyrA* gene corresponding to an aspartic acid to tyrosine amino acid change for nalidixic acid and ciprofloxacin resistance. The detected plasmid replicons indicated the presence of a SE serovar-specific pSEV plasmid, a ~60 kb, non-conjugative virulence plasmid that contains both the IncFII(S)_1 and IncFIB(S)_1 incompatibility regions (49), which was confirmed by PCR (data not shown). Nine virulence genes were plasmid-borne based on screening of the plasmid assembly. These included *pefA-D, spvB-C, spvR*, and *rck*. No AMR genes were detected on the pSEV plasmid. SE_TAU19 matched 1,127 pathogenic families compared to only two matched non-pathogenic families and was therefore predicted by PathogenFinder to have a 94.2% probability of being a human pathogen.

**Table 1:** Summary of antimicrobial resistance genes, plasmid replicons, and virulence genes detected in the draft genome sequence of *Salmonella* Enteritidis strain SE_TAU19 using ABRicate.

**Category**	**Gene(s)**	**Function**	**Accession number** ^a^
Antimicrobial resistance genes	*aac(6')-ly*	Chromosomal aminoglycoside resistance	AF144880:3542-3980
	*golS*	Gold-induced MdsABC efflux pump regulator	NC_003197:400860-401325
	*mdsA-C*	Mds multidrug and metal efflux pump	NC_003197.2:397058-398285
	*mdtK*	Multidrug and toxic compound extrusions transporter	CP014358.1:2161326-2162751
	*gyrA*	Fluroquinolone resistance (point mutation^b^)	None
Plasmid replicons	IncFII(S)_1	Virulence plasmid replicons	CP000858
	IncFIB(S)_1		FN432031
Virulence genes	*avrA; invA-C,E-J; orgA-C; pipB,B2; prgH-K; sifA-B; sipA-D; sopA-E; spaO-S; spiC; sptP; ssaC-D,G-V; sscA-B; sseA-K; sspH2; steA-C*	Type III secretion system	NP_461786; NP_461817; NP_461791; NP_460061; NP_461795; NP_460194; NP_461803; NP_461011; NP_461812; NP_460358; NP_461799; NP_460359; NP_460364; NP_460362; NP_461184; NP_460542
	*csgA-G*	Curli production, assembly, and transport	NP_460115
	*fimC,D,F,H,I*	Type I fimbriae	NP_459540
	*lpfA-E*	Long polar fimbriae	NP_462541
	*mgtB-C*	Mg transport protein	NP_462662
	*mig-14*	Antimicrobial peptide resistance	NP_461708
	*misL*	Putative autotransporter	NP_462656
	*pefB,D*	Plasmid-encoded fimbriae	NP_490511
	*ratB*	Outer membrane protein	NP_461449
	*rck*	Complement resistance	NP_490501
	*sic*	Virulence chaperone protein	NP_461807
	*sodCl*	Superoxide dismutase precursor	NP_460019

*A threshold of 100% gene coverage and ≥98% identity was utilized for detected genes*.

a *In cases of multiple genes within the same operon, only the accession number of the first gene listed is shown*.

b *Point mutations were detected with the ResFinder version 4.1 online database (https://cge.cbs.dtu.dk/services/ResFinder/)*.

## Discussion

A SE strain, SE_TAU19, caused persistent and reoccurring septicemic disease in two independent flocks of broiler chickens and was characterized using genomic analysis. This study revealed that the quinolone and sulfonamide resistant SE_TAU19 strain was lethal to broiler embryos and harbored a pSEV plasmid and typical SE virulence genes. SE_TAU19 was presumed to be vertically transmitted in the present study as is common for this pathogen (15). However, vertical transmission was not verified and existence of an on-farm reservoir host (e.g., rodents) was not eliminated. Genomic analysis of the pathogen was warranted due to septicemic disease severity, reoccurrence of disease in a subsequent broiler flock, and potential public health implications.

**Figure 3:**
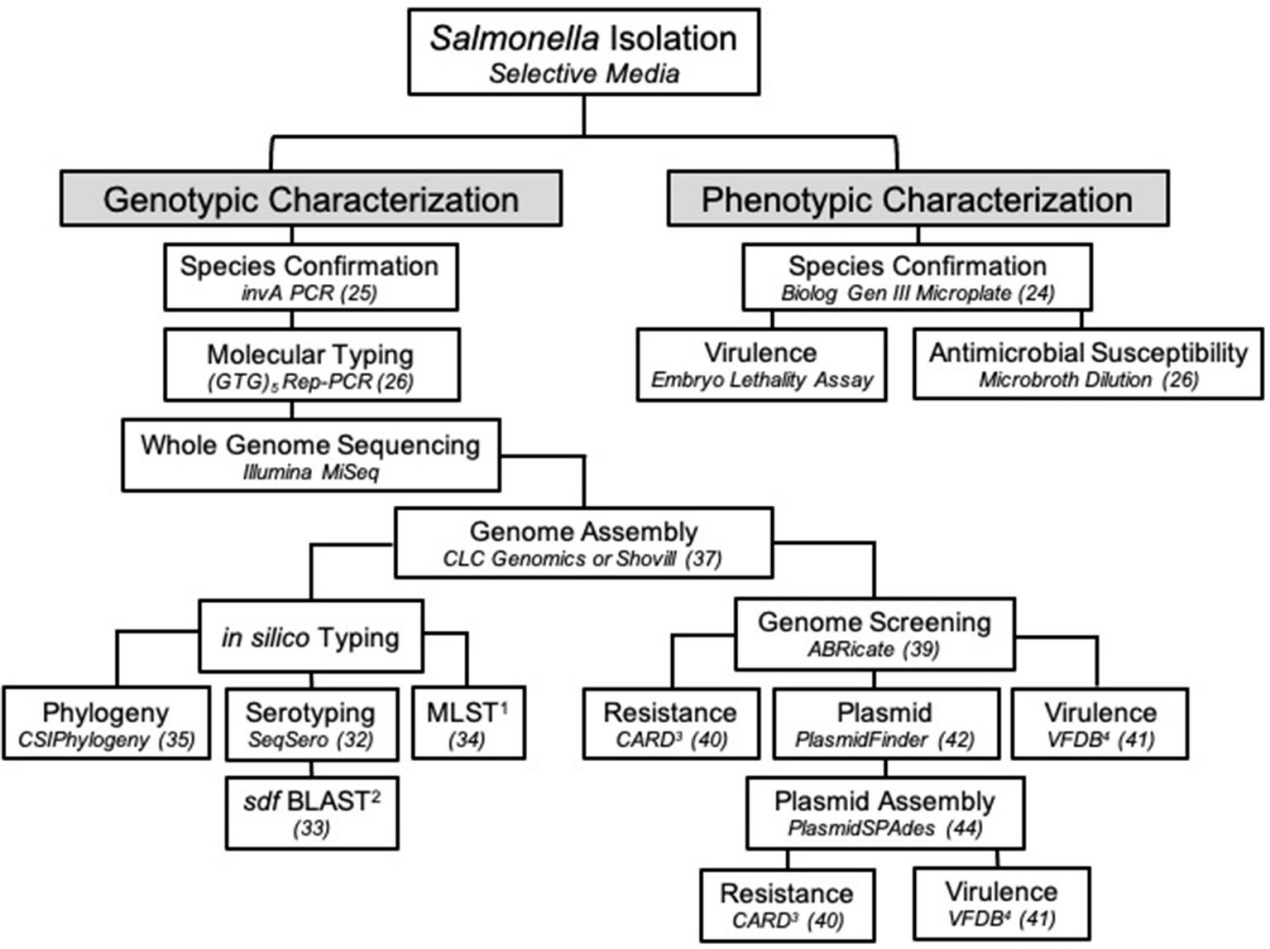
The workflow used to characterize *Salmonella* Enteritidis strain SE_TAU19 isolated from broiler chickens in this study. Specific methods or software and/or the corresponding reference are indicated in italics for each step. ^1^MLST, multilocus sequence typing; ^2^BLAST, basic local alignment search tool; ^3^CARD, comprehensive antibiotic resistance database; ^4^VFDB, virulence factor database.

The SE_TAU19 serotype was determined using the assembled genome and the SeqSero online serotyping by WGS tool (32). However, this tool cannot accurately differentiate *S*. Gallinarum from SE due to a non-expressed *fliC* allele in *S*. Gallinarum (32). Detection of the *sdf* fragment, which is specific to circulating strains of SE but not *S*. Gallinarum (33), in combination with MLST, is needed to distinguish between *S*. Gallinarum and SE when relying solely on *in silico* serotyping methods (Figure 3). Confirmation of SE by detection of the *sdf* fragment is especially important when serotyping *Salmonella* isolated from poultry by WGS as *S*. Gallinarum, the agent of fowl typhoid, is a reportable disease in the United States (50).

SE_TAU19 was resistant to nalidixic acid and sulfadimethoxine. Nalidixic acid resistance was corroborated by detection of the *gyrA* mutation in our genomic analysis (Table 1). Ciprofloxacin resistance was also predicted by this genotype, but the strain was designated as susceptible based on a MIC of 0.25 ng/μl. Quinolones such as nalidixic acid and ciprofloxacin are widely used first-line therapeutics for humans with salmonellosis when antimicrobial therapy is warranted (51), and increasing incidences of therapeutic failure in treating salmonellosis with ciprofloxacin have resulted in requests to re-evaluate human breakpoints for this drug (52, 53). Nevertheless, nalidixic acid resistant SE strains like SE_TAU19 have emerged in the southeastern United States (54) and elsewhere (55) contributing to the public health threat of drug-resistant, foodborne SE. Genetic determinants of sulfadimethoxine resistance in this strain were inconclusive, as AMR gene screening did not indicate a gene corresponding to this phenotype.

*In vivo* virulence of SE_TAU19 was investigated in an embryo lethality assay. SE_TAU19 achieved a 100% mortality rate compared to a virulent APEC strain which achieved an 80% mortality rate (Figure 2). SE_TAU19 virulence to broiler embryos was found to be comparable to published embryo lethality in *Salmonella enterica* serovar Gallinarum infections (56). This approach may be useful for future SE virulence or vaccine development studies. Additionally, the embryo lethality assay was a relatively simple means to investigate *in vivo* virulence potential and corroborate virulence gene discoveries. The contributions of specific SE_TAU19 genes to virulence in chickens would be best determined by deletion and complementation of the desired genes in SE_TAU19 followed by challenging SPF embryos and/or chicks.

This study highlights the utility of WGS in *Salmonella* identification and surveillance. The value of this approach has been realized through recent epidemiological advances, such as the ability to predict foodborne outbreaks of SE (20). The threat of zoonotic transmission of SE_TAU19 was supported when an *in silico* virulence prediction model PathogenFinder indicated that SE_TAU19 is likely a human pathogen due to sequence hits for several well-known SE virulence genes. Additional genomic analyses of pathogens can be used to improve the identification, prevention, and research of infectious agents that diminish food animal production and threaten public health. In this case, the characterization of SE_TAU19 could aid in the detection of clones in the broiler supply chain (26) or the development of SE autogenous vaccines (57). At the time of publication, there were over 29,000 SE genome assemblies available in the NCBI Sequencing Read Archive (SRA) database (58). However, most are yet to be characterized, and published pathogen genomes rarely include clinical details regarding the nature of disease caused in livestock. In Figure 3, we present a workflow for characterizing SE isolates that encompasses genotypic and phenotypic traits. This approach can be modified as appropriate for other *Salmonella* serovars and similar bacterial pathogens (e.g., *E. coli*).

In conclusion, the present study presents two separate instances of septicemic disease in broiler chickens which were attributed to SE strain SE_TAU19 based on genetic relatedness. Genomic analysis corroborated phenotypic resistance to antimicrobials and identified multiple virulence genes that may enhance its fitness as a poultry pathogen. However, further investigation is required to determine the biological relevance of these findings. Additional characterization of virulent SE strains is warranted, and this approach can be used for future studies.

## Data Availability Statement

The datasets presented in this study can be found in online repositories. The names of the repository/repositories and accession number(s) can be found below: https://www.ncbi.nlm.nih.gov/, AADSUW000000000.1.

## Ethics Statement

The animal study was reviewed and approved by NC State University Institutional Animal Care and Use Committee.

## Author Contributions

GW, MS, RC, and LB conceived and designed the experiments. DH and ST assisted with genomic data collection and analysis. GW, MS, SG, FJ, and LC were responsible for animal husbandry, post-mortem examinations, and sample collection. All authors reviewed and approved the final version of the manuscript.

## Conflict of Interest

The authors declare that the research was conducted in the absence of any commercial or financial relationships that could be construed as a potential conflict of interest.

## Publisher's Note

All claims expressed in this article are solely those of the authors and do not necessarily represent those of their affiliated organizations, or those of the publisher, the editors and the reviewers. Any product that may be evaluated in this article, or claim that may be made by its manufacturer, is not guaranteed or endorsed by the publisher.
